# Large-scale Plasma Proteomic Profiling Unveils Novel Diagnostic Biomarkers and Pathways for Alzheimer’s Disease

**DOI:** 10.21203/rs.3.rs-5167552/v1

**Published:** 2025-03-18

**Authors:** Carlos Cruchaga, Gyujin Heo, Alvin Thomas, Erming Wang, Hamilton Oh, Muhammad Ali, Jigyasha Timsina, Soomin Song, Menghan Liu, Katherine Gong, Daniel Western, Yike Chen, Patsy Kohlfeld, Allison Flynn, Joseph Lowery, John Morris, David Holtzman, Joel Perlmutter, Suzanne Schindler, Bin Zhang, David Bennett, Tammie Benzinger, Tony Wyss-Coray, Laura Ibanez, Yun Ju Sung, Ying XU, Patricia Moran Losada, Federica Anastasi, Armand Gonzalez-Escalante, Raquel Puerta, Natalia Vilor-Tejedor, Marc Suárez-Calvet, Pablo Garcia-Gonzalez, Maria Fernández, Mercè Boada, Amanda Cano, Agustín Ruiz

**Affiliations:** Washington University, School of Medicine; Washington University Medical School; Washington University, School of Medicine; Icahn School of Medicine at Mount Sinai; Stanford University; Washington University School of Medicine; Department of Psychiatry, Washington University School of Medicine, St. Louis, MO, USA; Washington University; Washington University; Washington University; Department of Psychiatry, Washington University School of Medicine, St. Louis, MO, USA; Washington University; Washington University School of Medicine, St Louis, MO, USA; Washington University; Washington University; Knight Alzheimer Disease Research Center; Washington University in St Louis School of Medicine; Washington University School of Medicine; Washington University School of Medicine; Icahn School of Medicine at Mount Sinai; Rush University Medical Center; Washington University in St. Louis; Stanford University; Washington University in St. Louis; Washington University Medical School; Washington University Medical School; Stanford University; BarcelonaBeta Brain Research Center; Barcelona Beta; Ace Alzheimer Center Barcelona – Universitat Internacional de Catalunya. Universitat de Barcelona (UB); BarcelonaBeta Brain Research Center; BarcelonaBeta Brain Research Center; Ace Alzheimer Center Barcelona; Washington University School of Medicine; Universitat Internacional de Catalunya; Ace Alzheimer Center Barcelona; Ace Alzheimer Center Barcelona, Universitat Internacional de Catalunya, Barcelona, Spain

## Abstract

Alzheimer disease (AD) is a complex neurodegenerative disorder. Proteomic studies have been instrumental in identifying AD-related proteins present in the brain, cerebrospinal fluid, and plasma. This study comprehensively examined 6,905 plasma proteins in more than 3,300 well-characterized individuals to identify new proteins, pathways, and predictive model for AD. With three-stage analysis (discovery, replication, and meta-analysis) we identified 416 proteins (294 novel) associated with clinical AD status and the findings were further validated in two external datasets including more than 7,000 samples and seven previous studies. Pathway analysis revealed that these proteins were involved in endothelial and blood hemostatic (ACHE, SMOC1, SMOC2, VEGFA, VEGFB, SPARC), capturing blood brain barrier (BBB) disruption due to disease. Other pathways were capturing known processes implicated in AD, such as lipid dysregulation (APOE, BIN1, CLU, SMPD1, PLA2G12A, CTSF) or immune response (C5, CFB, DEFA5, FBXL4), which includes proteins known to be part of the causal pathway indicating that some of the identified proteins and pathways are involved in disease pathogenesis. An enrichment of brain and neural pathways (axonal guidance signaling or myelination signaling) indicates that, in fact, blood proteomics capture brain- and disease-related changes, which can lead to the identification of novel biomarkers and predictive models. Machine learning model was employed to identify a set of seven proteins that were highly predictive of both clinical AD (AUC > 0.72) and biomarker-defined AD status (AUC > 0.88), that were replicated in multiple external cohorts as well as with orthogonal platforms. These extensive findings underscore the potential of using plasma proteins as biomarkers for early detection and monitoring of AD, as well as potentially guiding treatment decisions.

## Introduction

Alzheimer disease (AD) is the most common cause of dementia, contributing to 60–80% of dementia cases^1^. While it primarily affects older individuals, with symptoms typically appearing after the age of 65 (late-onset AD), about 1–5% of the cases have symptoms before 65 (early-onset AD). This progressive neurodegenerative disorder is characterized by the accumulation of amyloid-beta (Aβ) plaques, neurofibrillary tangles consisting of hyperphosphorylated and aggregated tau protein, and widespread neuronal loss. These pathological changes lead to cognitive decline, memory loss, behavioral changes, and ultimately, a loss of independence and functioning. The levels of these proteins (Aβ40, Aβ42, phosphorylated tau (ptau)181, ptau217, and specific tau peptides (MTBR-243)) have been shown to be reliable cerebrospinal fluid (CSF) and plasma biomarkers^2–8^ that capture amyloid pathology in the brain^9,10^, and tau pathology as measured by tau PET scans^8^.

AD is a complex and multifactorial condition, influenced by genetic, environmental, and lifestyle factors. Despite extensive research efforts, the exact mechanisms underlying the development and progression of AD remain incomplete. In recent years, proteomic studies have been instrumental in shedding light on the molecular complexities involved in AD. By analyzing proteins present in biological fluids and tissues, such as plasma, CSF, and brain tissue, proteomic studies^11–16^ have identified several protein biomarkers associated with AD. These biomarkers hold promise for early detection, accurate diagnosis, and monitoring of the progression. Furthermore, proteomic studies have the potential to unravel the underlying mechanisms and pathways contributing to AD pathology.

Although most of the proteomic studies in AD have been focused on the brain or CSF^11–13,17,18^, a handful of studies were also performed in plasma. Previous plasma proteomic studies in AD using Mass Spectrometry, Olink, or SomaScan have analyzed around 150–300 AD cases and controls^14,19–22^. Recent studies in larger cohorts utilizing SomaScan by Walker, *et al.*^23^ and Sung, *et al.*^15^ have identified tens of proteins in plasma associated with AD. Walker, *et al.*^23^ considered a population-based design with more than 4,000 individuals, but only 400 dementia patients were included on the analyses. They identified 38 proteins associated with the risk of developing AD. These proteins were part of the lipid, immunity, metabolic signaling, and hemostasis pathways. These proteins showed low to moderate predictive power with area under the curve (AUC) of 0.66–0.75. In another study, Sung, *et al.*^15^ generated plasma proteomic data (SomaScan) in an AD cohort and identified 26 proteins that showed a predictive power comparable to CSF Aβ and tau (AUC = 0.79). These proteins were enriched for programmed cell death and endolysosomal dysfunction. There are other studies that have performed proteomic analyses in plasma using Olink. Guo, *et al.*^24^ conducted a study utilizing data on 1,463 plasma proteins from over 50,000 individuals in the UK Biobank, but only 691 AD cases. They identified 16 proteins, including GFAP, NEFL, and GDF15, associated with incident AD that could be leveraged as biomarkers for predicting the incidence of dementia. Other Olink-based studies^25,26^ have identified between 33 to 429 proteins associated with AD but those had limited sample size (n < 1,200 and AD cases < 200). These and other studies are just a few examples of how plasma proteomic studies in AD have been instrumental in identifying potential biomarkers for AD as well as identifying new pathways implicated in the disease. As these studies have generally included a relatively small number of AD cases or general dementia, there is a need for a large-scale plasma proteomic study using well-characterized AD patients.

In this study, to address this need, we comprehensively examined 6,905 plasma proteins in 1,270 deeply phenotyped AD and 2,096 cognitively normal individuals, including a notably larger group of AD subjects compared to previous studies. We identified a robust set of plasma proteins associated with clinical AD status through rigorous stages and additional validations. We subsequently identified related pathways and novel biomarkers for predicting clinical AD and monitoring disease progression.

## Results

### Study Design

**Figure 1:**
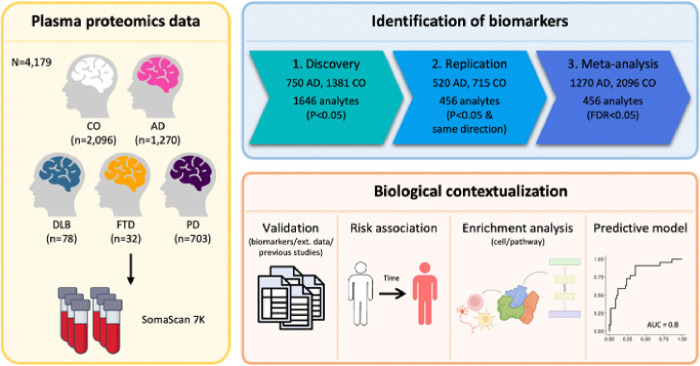
Study Overview. Plasma samples from the Knight ADRC and Stanford ADRC cohorts were analyzed using the SomaLogic SomaScan assay, measuring a total of 6,905 analytes targeting 6,106 proteins. Differential abundance analyses were conducted to compare cognitively normal individuals with those diagnosed with Alzheimer’s disease. A meta-analysis was then performed using results from both discovery and replication datasets. Identified proteins were further validated using Alzheimer’s disease biomarkers, external datasets and findings from previous studies. Subsequent analyses assessed the risk of progression to Alzheimer’s disease. Enrichment tests were conducted to explore associations with cell types and pathways. Lastly, a predictive model was trained to evaluate the performance of the selected proteins. Abbreviations: CO, Control; AD, Alzheimer’s disease; DLB, Dementia with Lewy bodies; FTD, Frontotemporal dementia; PD, Parkinson’s disease; Ext. data, External dataset.

We adopted a three-stage analytic framework to identify plasma proteomic signatures for AD and pathways implicated in AD pathogenesis and to develop a predictive model. In a discovery dataset of 2,131 samples (750 AD cases and 1,381 controls [CO]) from the Knight Alzheimer’s Disease Research Center (ADRC), we identified proteins associated with clinical AD status. These associations were replicated in an additional 1,235 samples (520 AD cases and 715 controls) from the Knight ADRC and Stanford ADRC cohorts (Fig. 1, Table 1). We performed meta-analyses using the results from discovery and replication dataset. The selected proteins were further validated using two external datasets from the Religious Orders Study and Rush Memory and Aging Project (ROSMAP; 150 AD and 322 CO) and Global Neurodegeneration Proteomics Consortium (GNPC; 1,733 AD and 4,833 CO). To determine the novelty of these protein associations, we compared our findings with those reported in plasma by Walker, *et al.*^23^, Sung, *et al.*^15^, Sattlecker, *et al.*^27^, Jiang, *et al.*^25^, Whelan, *et al.*^26^, Guo, *et al.*^24^ and in CSF by Ali, *et al.*^16^. As AD status was based on clinical diagnosis and cognitive tests, cases may include non-AD dementia, and controls may include preclinical AD. Therefore, we performed additional sensitivity analyses by comparing our results with those based on AD biomarker status (assessed through CSF Aβ and tau, amyloid imaging, or plasma ptau217; **Supplementary Table 1**).

To identify relevant biological processes, functions, and networks, we subsequently performed pathway and network analyses of the AD-associated plasma proteins. Then, we developed a machine learning-based classifier to predict the clinical status of AD. We further assessed its predictive power to predict biomarker status, progression from presymptomatic to symptomatic AD, and rate of memory decline. Additionally, we replicated and orthogonal validated the predictive model in additional datasets generated from other platforms, such as Alamar and Olink. Finally, by examining the predictive model for samples in dementia with Lewy bodies (DLB), frontotemporal dementia (FTD), and Parkinson’s disease (PD), we evaluated whether our model was specific to AD.

### Plasma proteins dysregulated with clinical AD

**Figure 2:**
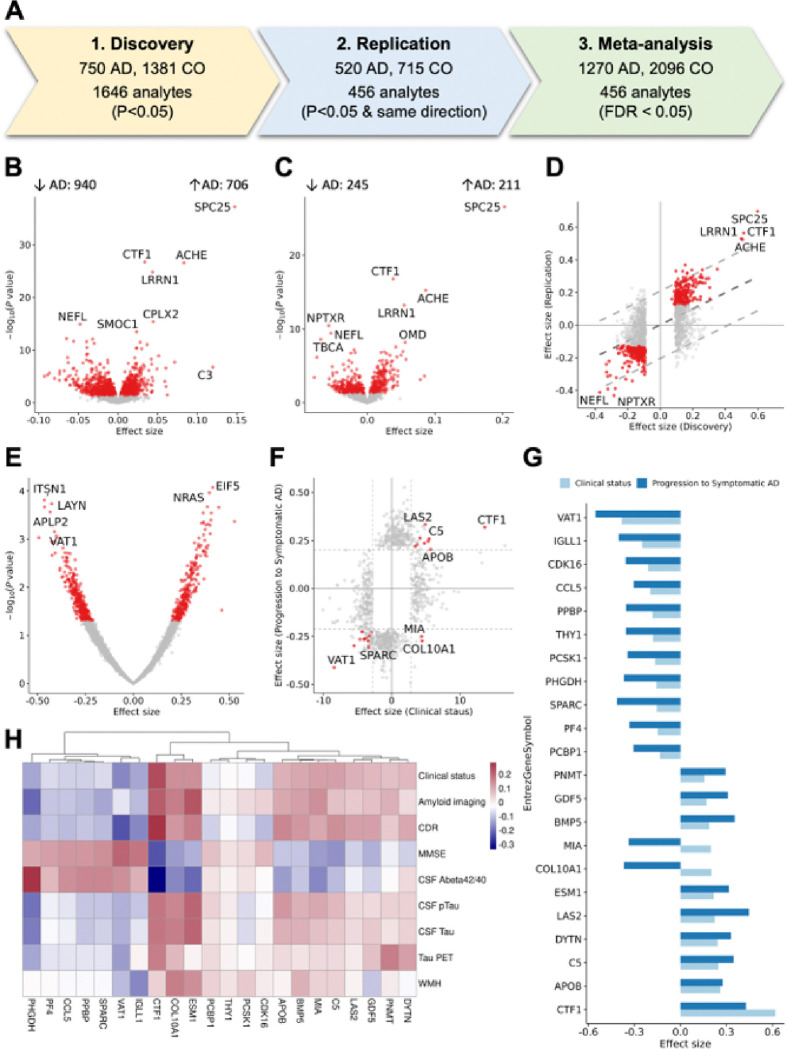
Differential Abundance Analysis. **(A)** Overview of the process for identifying plasma biomarkers dysregulated in clinical Alzheimer’s disease. **(B, C)** Volcano plots displaying proteins with significantly different abundances when comparing cognitively normal controls (CO) and Alzheimer’s disease (AD) patients in **(B)** Discovery and **(C)** Replication datasets. Red dots indicate proteins with a p-value < 0.05. **(D)** Scatter plot comparing effect sizes between the Discovery and Replication datasets, focusing on aptamers that were significant in the Discovery dataset (1,646 aptamers). The central dotted line shows the comparison pattern, with outer dotted lines marking the 95% confidence interval. **(E)**Volcano plot showing proteins with significantly different abundances when comparing individuals who converted to AD. **(F)** Scatter plot comparing effect sizes from the meta-analysis and AD risk analysis. Red dots indicate proteins that were significant in both analyses (FDR in meta-analysis and p < 0.05 in AD risk analysis) **(G)** Bar plot comparing effect sizes from the AD risk and clinical status analyses for 22 analytes significant in both the meta-analysis and AD risk analysis. **(H)** Heatmap showing correlations between 22 aptamers and AD-related phenotypes. Abbreviations: CO, Control; AD, Alzheimer’s disease; CSF, Cerebrospinal fluid; pTau, Phosphorylated tau; CDR, Clinical Dementia Rating; WMH, White matter hyperintensities; MMSE, Mini-Mental State Examination; Abeta, amyloid beta.

We quantified 6,905 plasma proteins (SomaScan 7K) and conducted a three-stage analysis to identify plasma proteins that exhibited significant differences between cognitively normal individuals and individuals with clinical AD (Fig. 2). The SomaScan assay is an indirect measure of protein levels, in which specific aptamers bind to the targeted proteins. However, for simplicity, we will refer to proteins instead of mentioning that each aptamer binds to specific proteins. Demographics of the cohorts and proteomic quality control (QC) procedures are in Materials and Methods. Associations were identified using regression of clinical AD on normalized Z-score protein levels^28^, including age and sex as covariates. In the discovery stage, we identified 1,540 proteins (1,646 aptamers) that were nominally associated with clinical AD status (Table 1, **Supplementary Table 2**). Among these proteins, 416 proteins (456 aptamers) showed nominal association in the replication stage with the same direction as the discovery data (**Supplementary Table 3**). In the meta-analysis, all 416 proteins (456 aptamers) passed false discovery rate (FDR) correction, based on a total of 6,905 aptamers (**Supplementary Table 4, 5, Supplementary Fig. 1**).

As the effect size for the associated proteins may be difficult to interpret, as it is based on normalized z-scores, we calculated the odds ratio (OR) for the 456 aptamers associated with AD risk by comparing the highest (3rd) and lowest (1st) tertiles of protein levels **(Supplementary Table 6A)**. This analysis showed that proteins with positive effect size had a mean OR of 1.50 (SD = 0.30), while proteins with negative effect size had a mean OR of 0.72 (SD = 0.08; **Supplementary Fig. 2).** On average, the proteins associated with AD risk identified in this study are associated with a 45% change on AD risk (mean of transformed OR = 1.45). Notably, the maximum OR observed was 3.58, highlighting a strong association between specific proteins and AD risk (**Supplementary Table 6B**).

### Replication in external datasets

**Figure 3:**
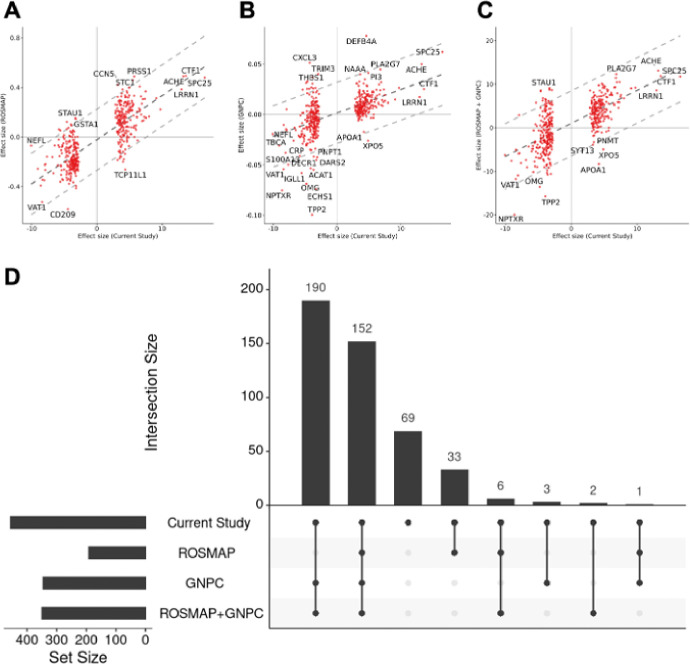
Replication in External Datasets. **(A-C)** Scatter plots comparing effect sizes from the meta-analysis with those from **(A)** ROSMAP, **(B)** GNPC, and **(C)** a combined meta-analysis of ROSMAP and GNPC, focusing on 456 proteins identified as significant in clinical Alzheimer’s disease. The central dotted line shows the comparison pattern, with outer dotted lines marking the 95% confidence interval. **(D)** Upset plot showing the overlap of significant analytes from the current study, ROSMAP, GNPC, and the combined meta-analysis with ROSMAP and GNPC, highlighting the 456 significant proteins.

To further validate our findings, we analyzed plasma proteomics (SomaScan 7K) data from the ROSMAP study (322 AD and 150 CO; **Supplementary Table 7A**). Of the aptamers identified in our study, 99% (453 aptamers) were present in the ROSMAP dataset, and 88% had a consistent direction of effect sizes (p = 2.89×10^−67^; Fig. 3A, **Supplementary Table 7B, 7C**). The correlation of effect sizes across these 453 aptamers was 0.793 (p = 4.02×10^−99^). In addition, 42% of the aptamers (192 aptamers) were nominally significant.

Additionally, we leveraged data from the GNPC (data freeze 1.1FF), a large collaborative study that includes plasma proteomics (SomaScan 7K) from 1,733 AD cases and 4,833 controls (**Supplementary Table 7A**). All significant aptamers identified in this study (n = 456) were present in the GNPC. Among these, 78% (354 aptamers) showed consistent direction (p = 6.19×10^−34^; Fig. 3B, **Supplementary Table 7B, 7C**) and showed a strong effect size correlation with Pearson correlation of 0.616 (p = 2.13×10^−40^) when considered nominally significant aptamers. A total of 75% (346 aptamers) of the 456 identified aptamers were nominally significant in GNPC, and 72.5% (331 aptamers) passed multiple testing correction.

Subsequently, we performed a meta-analysis combining results from the ROSMAP and GNPC datasets. Of the 456 significant aptamers, 78% (353 aptamers) demonstrated consistent direction of effect sizes (p = 1.90×10^−34^; Fig. 3C, **Supplementary Table 7B, 7C**). In this meta-analysis, 77% (350 aptamers) were nominally significant, and 74% (333 aptamers) remained significant after multiple testing correction. The correlation of effect size for 453 aptamers that were present in the meta-analysis was 0.675 (p = 1.33×10^−66^). Taken all this together, of the 456 aptamers that were present on the ROSMAP or the GNPC, a total of 387 aptamers (85%) showed nominal association in one of the studies or the meta-analyses (Fig. 3D). Notably, among 456 aptamers, 312 exhibited consistent effect size directions across, ROSMAP, GNPC, and ROSMAP-GNPC meta-analysis, despite differences in cohort characteristics. These 312 aptamers include notable proteins such as SPC25, CTF1, ACHE, and CPLX2, which were among the most significant proteins identified.

### Comparison with previous plasma studies

We systematically compared our results to the previous findings from six plasma studies, three based on SomaScan: Walker, *et al.*^23^, Sung, *et al.*^15^, Sattlecker, *et al.*^27^; and three based on Olink: Jiang, *et al.*^25^, Whelan, *et al.*^26^, and Guo, *et al.*^24^ (**Supplementary Table 8A-D, Supplementary Fig. 3**). By examining SomaScan 5K data in 4,110 samples (428 dementia cases), Walker, *et al.*^23^ identified 38 proteins significantly associated with incident dementia, after correcting for multiple testing. Among the 456 aptamers (416 proteins) identified in our study, 319 aptamers were present in the result by Walker, *et al.*^23^ (**Supplementary Table 8B, 8D** and **Supplementary Fig. 3B**). Among those, three aptamers were reported to be significant after correction in the original study, 52 aptamers showed a nominal association, and 176 (55%) showed a consistent direction between the two studies. Overall, the 52 aptamers that were nominally significant in the study showed an effect size correlation with a Pearson coefficient of 0.872 (p = 4.16×10^−17^). In their study, all subjects were controls at the time of the plasma collection, among which several of them developed dementia (not just AD) between 5 to 25 years after plasma collection. Despite employing a survival analysis for incident dementia, there was a high correlation between the results from these two studies.

Sung, *et al.*^15^ analyzed plasma proteomics (SomaScan 1.3K) data from 105 AD cases and 254 controls and identified 26 proteins associated with AD after Bonferroni correction. Out of the 456 aptamers identified in our study, 67 aptamers were present in their result. Among these, two aptamers passed multiple test correction, 34 aptamers were nominally significant, and 52 (78%) showed consistent direction (**Supplementary Table 8B, 8D** and **Supplementary Fig. 3C**). Among 34 aptamers, aptamers that were nominally significant in the study^15^ exhibited a strong correlation, with a Pearson coefficient of 0.805 (p = 9.42×10^−09^).

Sattlecker, *et al.*^27^ analyzed plasma SomaScan data measuring 1,001 proteins from 331 cases and 211 controls, and identified 138 nominally significant proteins associated with AD, and four proteins after FDR correction. Out of the 456 aptamers identified in our study, 76 (84%) aptamers were present in their result. Among these, two aptamers were found to be significant after applying multiple testing correction, 22 aptamers were nominally significant, and 64 aptamers showed consistent direction in their study (**Supplementary Table 8B, 8D** and **Supplementary Fig. 3D**). The effect size correlation for aptamers that were nominally significant (n = 22) exhibited a correlation of 0.811 (p = 4.72×10^−09^).

Jiang, *et al.*^25^ analyzed plasma proteomics data using the Olink platform, measuring 1,160 proteins in 106 AD cases and 74 controls. They identified 429 proteins significantly associated with AD after FDR correction. In our study, 37 proteins overlapped with their findings, and 34 proteins (92%) exhibited consistent direction and passed multiple test correction. The correlation between effect sizes in the two studies was highly significant (Pearson = 0.669; p = 5.99×10^−06^; **Supplementary Table 8D and Supplementary Fig. 3E**).

Whelan, *et al.*^26^ analyzed plasma proteomics data using Olink, measuring 250 proteins from a cohort consisting of 557 cognitively normal individuals (Aβ + and Aβ−) and 161 Aβ + AD cases. They identified 33 proteins significantly associated with AD after FDR correction. Of the 416 proteins (456 aptamers) identified in our study, 22 overlapped with their results, showing significant effect sizes correlation (p = 3.63×10^−02^; **Supplementary Table 8D and Supplementary Fig. 3F**). Among these, 4 proteins passed multiple testing correction.

Guo, *et al.*^24^ leveraged the Olink platform to measure 1,463 proteins in 51,228 controls and 691 incident AD cases from the UK Biobank (UKBB). They identified 16 proteins associated with the risk of developing dementia after FDR correction. Similar to Walker, *et al.*^23^, they employed a Cox regression model for survival analysis. In our study, 122 proteins overlapped with their findings, among which 3 passed multiple testing correction. Importantly, 89 proteins (73%) demonstrated consistent direction (p = 4.04×10^−07^; **Supplementary Table 8D and Supplementary Fig. 3G**).

In summary, some of the identified proteins, SPARC-related modular calcium-binding protein 1 (SMOC1), complexin-2 (CPLX2), spondin-1 (SPON1), neurofilament light polypeptide (NFL), or alpha-1-antichymotrypsin complex (SERPINA3), have been reported in earlier plasma proteomic studies or are known AD biomarkers (e.g., NFL). Several proteins, including kinetochore protein Spc25 (SPC25), cardiotrophin-1 (CTF1), acetylcholinesterase (ACHE), and leucine-rich repeat neuronal protein 1 (LRRN1) had not been reported to be associated with AD or dementia in recent large-scale plasma proteomics studies^15,23–27^, although they were identified in a recent large-scale CSF proteomic study^16^. Lastly, other proteins such as dehydrogenase/reductase SDR family member 9 (DHRS9), D-3-phosphoglycerate dehydrogenase (PHGDH), and islet amyloid polypeptide (IAPP), have not been reported in any previous plasma^15,23–27^ or CSF^16^ studies.

Taken all this together, out of the 416 proteins (456 aptamers) identified in our current study, 334 have been analyzed on other plasma studies^15,23–27^ (**Supplementary Table 8D**). Of these, 122 (37%) proteins have been reported to be nominally significant and, of those 52 (16%) were significant after multiple test correction in at least one of the previous plasma studies. The remaining 212 proteins, even included in other studies, have not been reported associated with AD risk. This difference may be due to smaller sample size and/or different study design. As this could be due to statistical power, as most of the previous plasma studies had lower sample size, we analyzed the effect size correlation as well as concordance in direction. A total 204 (61.1%) were in the same direction with previous studies, suggesting that in fact previous studies did not have enough statistical power. A significant effect size correlation for all studies (p < 3.63×10^−2^), as well direction concordance (range 55–93%) and these findings is not platform-specific as similar concordance is found for both the SomaScan- and Olink-based studies. These results support the robustness of our findings.

### Cross-tissue comparison

A recent large-scale CSF proteomics study (N = 2,286) identified 2,173 aptamers (2,029 proteins) associated with clinical and biomarker AD status^16^. This study was conducted using proteomics data from Knight ADRC cohort and used the same platform and a similar analytical approach, which allowed us to determine the overlap of proteomic signatures between CSF and plasma. Among the 456 aptamers identified in our study, 445 were present in the CSF study. We found that 174 aptamers were statically significant after multiple testing correction in CSF. The top three proteins in plasma (SPC25, CTF1, and ACHE) also showed strong association in CSF (p < 1.01×10^−3^), but these were not reported in the previous plasma studies. The effect size in CSF and plasma for these 445 aptamers showed a moderate correlation of 0.419 (p = 2.07×10^−20^). Of the top 100 plasma aptamers, 45 (45%) were significant in CSF, and of the top 200 plasma aptamers, 85 (42%) were significant in CSF.

Among the 2,173 aptamers (2,029 proteins) identified in the CSF study, 174 aptamers (164 proteins) were associated after multiple test correction in plasma (**Supplementary Table 8C, 8D, Supplementary Fig. 3A**). SMOC1, which ranked among the top 10 significant proteins in plasma study, has previously been identified as the most significant finding in CSF and was reported in previous studies by Sung, *et al.*^15^, Walker, *et al.*^23^, and Shen, *et al.*^29^. However, other top CSF proteins, such as YWHAG, TMOD2, YWHAB, PPP3R1, TMOD3, DLG2, YWHAZ, YWHAE, MAPRE3, ATP6V1F, or HOMER1, were not significant in plasma. In fact, of the top 20 CSF proteins, only two were significant in plasma (4 in the top 50; and 8 in the top 100). Only about 8% of the CSF-associated proteins showed association in plasma. Despite some overlap across tissues, proteomic signatures in CSF and plasma appear to be different, leading to a different set of proteins (and subsequently different biomarkers) and implying unique underlying biology.

Integrating the analyses comparing the proteins identified in this study with those identified in previous plasma and CSF studies that we compared, of the 456 aptamers, 193, including Clusterin (CLU) and FAHD2A, were novel and have not reported in any previous plasma or CSF study. Additionally, 140 aptamers (SPC25, CFT1, ACHE), hereinafter as “plasma-novel”, were not found in previous plasma studies, but reported to be associated with AD in CSF. There were 71 aptamers (i.e. MIA, CPLX1, and APOE), hereinafter as “plasma-nominal”, that have been reported to be associated with AD at nominal significance, not after multiple testing correction, in previous studies. There were 52 (i.e.: APOB, SERPINA3, or COL10A1, “plasma-significant”) that passed multiple test correction in previous studies.

### The identified proteins are also associated with AD biomarker-status

While this study focused on clinical status of AD, we performed sensitivity analysis using biomarker-based status and compared it with the clinical AD status to evaluate whether our identified proteins also capture underlying AD pathology. Biomarker status was defined using three different approaches: 1) amyloid/tau (AT) status based on Aβ42 and ptau181 values in CSF (105 A + T + vs. 256 A−T−), 2) amyloid PET imaging (122 A + vs. 265 A−), and 3) plasma ptau217^30^ (1,217 T + vs 942 T−; **Supplementary Fig. 4, Supplementary Table 9–12)**.

As the sample size was larger for the plasma ptau217, we found larger overlap of significant proteins (268 of the 456 aptamers) for this AD biomarker than for CSF AT or amyloid imaging status (**Supplementary Table 12**). However, in our analyses, amyloid imaging status showed the largest number of proteins (82%) with consistent effect size, as well as the highest effect size correlation (*p* = 0.764), followed by CSF AT status (74% of concordant effect size proteins and *p* = 0.705). The top eight proteins in our main analyses (including SPC25, CTF1, ACHE, LRRN1, NFL, CPLX2, SMOC1, TBCA) were also significant across all three biomarker analyses. In summary, for each of these analyses, we found strong correlations in the effect size (Pearson correlation > 0.7) and consistent direction of the association (**Supplementary Fig. 4, Supplementary Table 9–12)**.

### Identifying proteins associated with progression to symptomatic AD

Among the cognitively normal participants at blood draw included in this study, 761 had subsequent follow-up clinical assessments (3.5 ± 2.4 years) after plasma collection (maximum = 15 years; **Supplementary Table 13**). Of these, 83 individuals progressed to symptomatic AD (from CDR = 0 to CDR > 0 and clinical diagnosis of AD). We leveraged these data to identify proteins associated with progression to symptomatic AD. In the survival analysis with a Cox proportional hazards model, including age at blood draw and sex as covariates, we found 625 proteins being nominally associated with progression (**Supplementary Table 14** and **Supplementary Fig. 5**). Of the 456 aptamers associated with clinical status, 22 aptamers were also associated with progression to symptomatic AD. We found that 20 (out of these 22) had consistent effect size directions with the analysis of clinical AD status. The two proteins in the opposite direction were MIA and COL10A1. MIA is primarily expressed in melanocytes and Schwann cells and is involved in extracellular matrix organization and cell-matrix adhesion. COL10A1 is expressed in oligodendrocytes and neurons and is known to contribute to cell proliferation and migration.

### Pathways involved in plasma proteins

Next, we performed cell-type enrichment and pathway analyses for the 416 proteins (456 aptamers) that exhibited association with clinical AD to better understand the biological processes these proteins were involved in. Brain cell type enrichment analysis revealed that these 416 proteins were primarily enriched in oligodendrocytes (p = 3.67×10^−2^) and astrocytes (p = 1.86×10^−2^), and depleted in neuronal proteins (p = 7.54×10^−3^; **Supplementary Table 15**), suggesting that they reflect pathways related to axonal and overall brain health.

**Figure 4:**
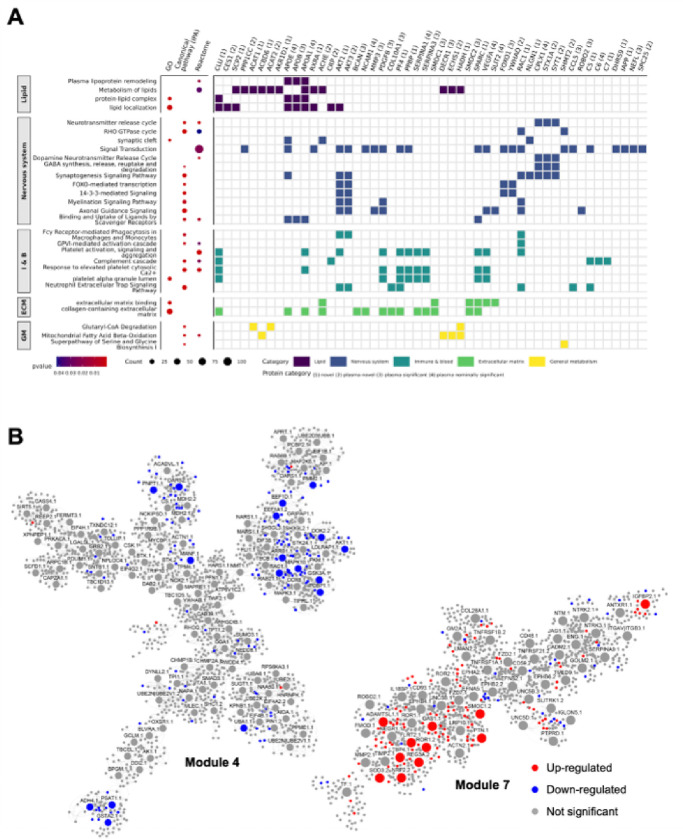
Pathway and Network Analyses of AD associated Proteins. **(A)**Heatmap showing the association of specific genes with biological pathways and processes from GO, IPA, and Reactome. The color coding corresponds to the specific group each pathway belongs to. **(B)** Networks of two key modules M4 and M7 identified through the MEGENA. Nodes represent individual proteins, with up-regulated proteins colored in red and down-regulated proteins in blue. Protein without significant changes in AD are shown in gray. Hub proteins are shown as larger nodes. Abbreviations: GO, Gene Ontology; IPA, Ingenuity Pathway Analysis.

Pathway analysis indicates that these plasma proteins are primarily involved into five main areas: 1) lipid metabolism; 2) immune and hemostasis 3) extracellular matrix; and 4) neuronal-related; 5) general metabolism pathways (Fig. 4A; **Supplementary Table 16–18, Supplementary Fig. 6**).

The most significant lipid-related pathways included transport (FDR p = 2.45×10^−3^), localization (FDR p = 4.20×10^−3^) and catabolism of lipids (FDR p = 2.12×10^−2^; **Supplementary Table 16**). These lipid processes include proteins that are already known to be part of the causal pathway of AD such as APOE, CLU and BIN1. Although APOE protein levels have been reported to be nominally associated with AD risk in previous plasma studies, this is the first time its association after multiple test correction in a proteomic study. CLU, a known protein involved in lipid metabolism, has not been reported to be associated with AD in either CSF or plasma in previous studies. SPP1, also known as osteopontin, is involved in lipid-associated macrophages mediated inflammation^31^ and has been associated with microglia activation and memory decline^32^. Although lipids metabolism is known to be part of AD pathogenesis, this study identified 40 novel proteins that have not been associated with AD previously and their involvement for this pathway, thereby extending previous findings. These proteins included SMPD1^33^, PLA2G12A^34^, CTSF^34^ or SPARC^35^ that have been reported to carry variants that increase risk for AD, neurodegeneration or affect blood-brain barrier.

Our analyses additionally identified pathways related to immune and hemostasis-related processes (**Supplementary Table 17**). Immune and inflammation pathways were driven by the novel proteins C5, CFB, DEFA5, FBXL4, and PPBP, as well as “plasma-novel” proteins, CRP and STX1A. They were part of the complement pathways (C5, C7, CFB) or the GPVI-mediated activation cascade (novel: CDC42, RAC1, RAC2) which is involved in immune response and more importantly in vascular integrity. Other pathways related to general integrity of the blood functions and hemostasis included elevated platelet cytosolic Ca2+ (p = 8.71×10^−10^; CLU, PF4, and PPBP), platelet activation, signaling and aggregation (p = 7.13×10^−5^; CDC42, CLU), and neutrophil extracellular trap signaling pathways (p = 1.78×10^−5^; COL10A1, ROBO2, and VEGFA).

Extracellular several matrix-related pathways were also identified: collagen-containing extracellular matrix (p = 3.55×10^−5^), extracellular matrix (p = 8.39×10^−7^), and extracellular matrix binding (p = 4.10×10^−5^) that are being driven by ACHE, SMOC1, SMOC2, VEGFA, SPARC, which also point to overall vascular health, together with VEGFA, and SMOC proteins are related to vascular endothelial cells. Although SMOC1, SMOC2, NCAM1 or VEGFA were among the most significant proteins associated with AD in CSF, additional novel endothelial and vascular related proteins were found to be associated with AD risk in plasma.

In addition to blood or immune response pathways, a large number of proteins were related to the brain and the central nervous system including axonal guidance signaling (p = 6.46×10^−5^, EIF4E, ROBO2 and VEGFA) and myelination signaling (p = 3.98×10^−3^, involving AKT1, AKT3, and BMP6). We also found 14–3-3-mediated signaling and FOXO-mediated transcription (p < 3.16×10^−5^; FOXO1, YWHAQ) along with several neuronal system pathways including synaptogenesis signaling (p = 1.02×10^−4^ involving FOXO1, YWHAQ, APOE, CPLX1, CPLX2 and STX1A), and GABA synthesis, release, reuptake, and degradation (p = 3.80×10^−3^; involving CPLX1, STX1A, and SYT1; **Supplementary Table 17**). Other top pathways included signal transduction pathway (p < 4.77×10^−2^, involving AKT1, AKT3, and RXRA) and those related to neurotransmitter release cycle (p = 8.50×10^−4^; involving SLITRK3, ARHGAP30, and ARHGAP45) such as RHOA GTPase cycle (p = 2.32×10^−2^) and RHO GTPase cycle (p = 4.19×10^−2^), involving SHMT2 and RAC1.

### Plasma Protein Co-Expression Network in AD

To further elucidate the complex interactions across all the 6,905 plasma proteins, we employed the Multiscale Embedded Gene co-Expression Network Analysis (MEGENA)^36^. In total, 274 modules were detected in the protein co-expressed network, with an average of 81 proteins, ranging from 10 to 1,590 (**Supplementary Table 19**).

Of the 274 modules, thirty-one modules were significantly enriched for the identified 456 aptamers (416 proteins) associated with the clinical status of AD. Modules M4 and M7 were the top-ranked modules based on aggregated Cauchy association test (ACAT) method^37^. The module M4, consisting of 1,519 proteins included hub proteins 11 “novel” proteins (UBA1, ADH4, or GSTA2), as well as plasma-novel (MAPK10) and “plasma-nominal” (GSK3A). This module was primarily enriched for the down-regulated proteins (mean effect size − 3.62; Fig. 4B). M4 was enriched in oligodendrocytes (p = 8.52×10^−2^) and involved in intracellular transport (p = 5.17×10^−17^, PPP1CC and YWHAQ, both “plasma-novel” proteins), translation (p = 8.20×10^−11^, involving AKT1 (novel) and EIF4E (novel)) and macroautophagy (p = 7.53×10^−10^, involving AKT1 and RAB1B (novel); **Supplementary Table 20**).

In contrast, consisting of 930 proteins, the M7 module included two “novel” proteins (ROR1 and REG3A) as well as SMOC1 (reported in CSF multiple times) as hub proteins. This module was enriched for up-regulated proteins (mean effect size 2.26; Fig. 4B). M7 was enriched in endothelial cells (p = 7.53×10^−10^, SMOC1, SMOC2, VEGFA COLEC12, and GPD1 among others) and neurons (p = 2.35×10^−3^, NPTXR; ROBO2 and CPLX2) and was enriched in extracellular matrix organization (p = 5.46×10^−13^, SMOC1 and SMOC2), axon guidance (p = 7.73×10^−13^, involving ROBO2 and VEGFA), and axonogenesis (p = 2.43×10^−12^, involving NRP2 and NRCAM), re-emphasizing the pivotal role of extracellular matrix and axonal processes in AD. In addition, this result supports several brain and neuronal pathways identified above for plasma proteins associated with AD (**Supplementary Table 20**).

### Seven plasma proteins with high AD-specific predictive power

**Figure 5:**
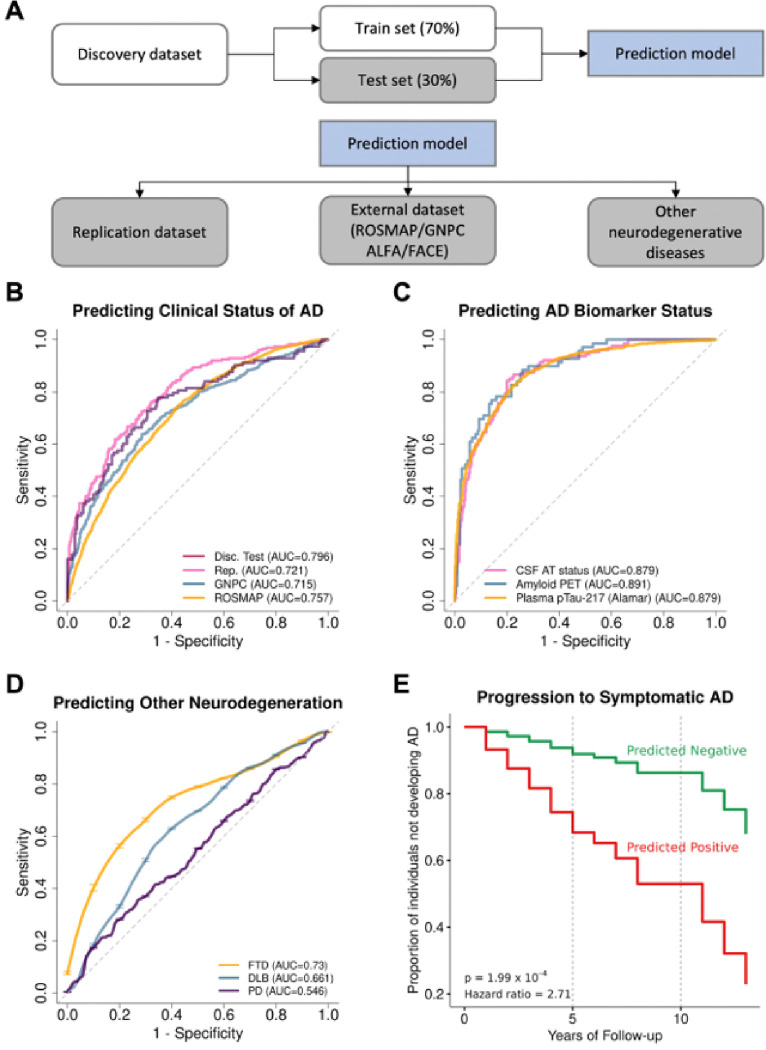
Predictive Performance of the Model with 7 Plasma Proteins. **(A)** Diagram showing how the datasets were used for training and testing the predictive model. **(B)** Receiver Operating Characteristic (ROC) curves evaluating the model’s predictive power in classifying clinical Alzheimer’s disease (AD) status using two independent datasets: the test set from Discovery dataset (red). Replication dataset (pink), GNPC (blue) and ROSMAP (orange). **(C)** ROC curves for distinguishing AD biomarker status, including CSF AT status (pink), Amyloid PET (blue), and ptau217(orange). **(D)** ROC curves for distinguishing frontotemporal dementia (FTD; orange), dementia with Lewy bodies (DLB; blue), and Parkinson’s disease (PD; purple). **(E)** Kaplan-Meier survival curves for progression to symptomatic AD after the initial blood draw. The predicted negative group (green line) and the predicted positive group (red line) are plotted, illustrating the proportion of participants remaining cognitively normal over years of follow-up. The p-value indicates the significant difference in progression to AD between individuals predicted to have AD versus controls, according to the cox proportional hazard model. Abbreviations: AD, Alzheimer’s disease; Disc, Discovery; Rep, Replication; CSF, Cerebrospinal fluid; AT, Amyloid/tau; PET, Positron emission tomography; pTau, Phosphorylated tau; FTD, Frontotemporal dementia; DLB, Dementia with Lewy bodies; PD, Parkinson’s disease.

Finally, we examined if the 456 aptamers (416 proteins) associated with AD could be leveraged to develop a predictive model for clinical diagnosis or biomarker status. We used the least absolute shrinkage and selection operator (Lasso) regression model to select proteins for predicting the clinical status including age and sex as covariates. We split the discovery dataset with 70% used for training the model (discovery-train; 967 cognitively normal and 525 AD participants) and the remaining 30% for validating the model (discovery-test). In addition, the replication dataset and external datasets (ROSMAP, GNPC, ALFA, and FACE) were also considered to examine predictive power of the model (Fig. 5A).

The discovery-training data was used to identify the minimum set of proteins that maintain a strong predictive power and to determine the cutoff value for prediction and weights for these identified proteins (**Supplementary Table 21**). This model (with the trained set of proteins, their weights, and prediction cutoff value) was then applied to examine their predictive power for clinical and biomarker status, as well as progression to symptomatic AD and rate of memory decline. The seven-protein (SPC25, CTF1, ACHE, CPLX2, VAT1, NPTXR and FAHD2A) predictive model resulted AUC for clinical status of 0.796 in the discovery-test and 0.72 in the replication, showing significantly better performance (p = 8.21×10^−10^) than the baseline model (AUC of 0.661, with age and sex; **Supplementary Table 22A, 22B**). Similar predictive power was observed for GNPC cohort (AUC of 0.715) and ROSMAP cohort (AUC of 0.757; Fig. 5B, **Supplementary Fig. 7**).

As cognitively normal individuals may include asymptomatic individuals that may underestimate the predictive power of these proteins, we applied this model to predict biomarker status based on amyloid status (based on amyloid imaging), plasma ptau217, or CSF Aβ and ptau181. In this analysis, the predictive power for this model was significantly higher than that for clinical status, showing an AUC of 0.88 for amyloid positivity, 0.89 for CSF amyloid/tau status, and 0.88 for plasma ptau217 status (Fig. 5C, **Supplementary Table 22A**).supporting the hypothesis that the lower AUC found for clinical status may be driven by the presence of asymptomatic individuals.

To further compare our predictive model with well-established AD biomarkers, we analyzed plasma ptau181, ptau217, Aβ40, Aβ42, NEFL, and GFAP measured using the Alamar/Nulisa platform^30^ in the same samples. The seven-protein model (AUC = 0.794) demonstrated comparable predictive power to ptau217 (AUC = 0.798) and surpassed Aβ40, Aβ42, NEFL, GFAP, and ptau181. (**Supplementary Table 23** and **Supplementary Fig. 8**). Plasma Alamar ptau217 showed an AUC of 0.94 against brain amyloid positivity, in line with the best-in-class plasma ptau217 assays^30^. These findings emphasize the robustness of our seven-protein signature, demonstrating its potential to perform on par with state-of-the-art biomarkers and further validating its clinical relevance, and confirming that the relatively low AUC for clinical status is likely driven by the presence of asymptomatic individuals.

For the purpose of further validate the predictive model findings, we employed additional platforms and cohorts. Specifically, we utilized the Alamar and Olink platforms to independently assess the predictive power of the identified proteins. Comparative analyses were conducted between SomaScan and Alamar data from the Knight-ADRC cohort (1,166 cases and 1,595 controls), as well as between SomaScan and Olink data from the Stanford ADRC cohort, ALFA cohort at Barcelona Beta Brain Research Center (BBRC) and the Fundacio ACE (FACE; **Supplementary Fig. 9–11, Supplementary Table 24**). Both single-protein and multi-protein model performances were tested. As none of the platforms included all seven proteins, we performed a head-to-head comparison between SomaScan vs Alamar or Olink for those proteins present on both platforms. The differences in AUC values for both single-protein and multi-protein models were less than 0.07.

Using Alamar data, we validated the ACHE and NPTXR combination and observed an AUC of 0.728 with Alamar-based protein measures and 0.704 based on SomaScan (1,166 AD cases and 1,595 controls from the Knight ADRC, **Supplementary Fig. 9, Supplementary Table 25**). We also analyzed SomaScan and Olink data generated from 94 cases and 596 biomarker confirmed controls from the Stanford ADRC cohort. The predictive power of the four proteins that were included on Olink (ACHE, NPTXR, CTF1, and VAT1) was 0.946 compared to 0.952 with SomaScan (**Supplementary Fig. 9, Supplementary Table 25**). We also performed additional analyses including all the Stanford ADRC Olink data, without the need to match SomaScan data samples. This increased the number of samples analyzed to 120 AD cases and 725 controls (**Supplementary Fig. 10**). The predictive power for these four proteins in this extended dataset was 0.874. The higher predictive power in the Stanford ADRC is likely due to the use of biomarker-confirmed AD cases cand controls. Similarly, we replicated the SomaScan seven-protein predictive model using ALFA cohort data from BBRC (AUC = 0.751; 132 cases and 260 CSF controls; **Supplementary Fig. 11**). Olink data from this cohort was also analyzed, for orthogonal validation. Similar to Stanford dataset, only ACHE, NPTXR, CTF1, and VAT1 were present on the ALFA cohort, we compared the SomaScan vs Olink predictive model including the four proteins, observing comparable predictive power between Olink (AUC = 0.657) and SomaScan (AUC = 0.695; **Supplementary Fig. 11**). Finally, similar analyses were performed on Olink data generated on 506 serum samples and Somalogic data on 795 plasma samples from the FACE cohort. The AUC for the single proteins model varied from 0.846 to 0.897 for the Olink-based measures and 0.846 to 0.885 for the SomaScan-based data (**Supplementary Fig. 11**). These independent validation efforts confirmed the consistency and robustness of the prediction model across platforms, even though we could not test a model with all seven proteins **(Supplementary Fig. 9, Supplementary Table 25).**

To test if the seven-proteins predictive model can also identify cognitively normal individuals that will progress to symptomatic AD, we examined whether this model could identify individuals who progress from cognitively normal to symptomatic AD. We analyzed data from 761 individuals who were cognitively normal at the time of plasma draw, of whom 83 eventually progressed to symptomatic AD. Using a Cox proportional hazard model, we found that individuals who were predicted positive based on the seven-protein model (i.e., predicted as AD) had a 2.7 times higher risk of developing symptomatic AD compared to the predicted negative group (hazard ratio = 2.71; p = 1.99×10^−4^; Fig. 5E, **Supplementary Fig. 12**). When analyzing the prediction performance for risk of developing to symptomatic AD in 5, 10, or 15 years, this seven-protein model showed an AUC ranging from 0.71 to 0.73 (**Supplementary Fig. 12)**.

We also investigated if this seven-protein model could also have the power to predict rate of memory decline. To assess this, we considered 1,672 individuals who had longitudinal follow-up information with Clinical Dementia Rating^®^ sum of boxes (CDR^®^-SB). This analysis revealed that those individuals who were positive (predicted as AD) for the seven-protein signature exhibited a significantly faster rate of memory decline (slope of CDR-SB/year) than those negative (predicted as CO) for the seven-protein signature (**Supplementary Table 26, Supplementary Fig. 13**). Specifically, approximately 1 point difference of CDR-SB per year, was observed between the two predicted groups (1.40 vs. 0.39; p = 1.95×10^−64^), when comparing all individuals regardless of the diagnosis at blood draw. The rate of change in CDR-SB was further analyzed in individuals who were cognitively normal (CO) or had AD at the time of the blood draw. Cognitively normal individuals who predicted positive for the seven-protein signature exhibited significantly higher CDR-SB changes (slope = 0.46) compared to those who tested negative for this model (slope = 0.09; p = 2.84×10^−21^). Among AD cases, those positive for the seven-protein signature also showed higher CDR-SB/year slope than those who were negative (2.02 vs 1.67 per year, respectively; p = 1.85×10^−2^).

Finally, we evaluated whether this model is specific to AD or whether it possesses some ability to predict dementia caused by other neurodegenerative diseases (Table 2). The AUC for predicting PD and DLB was 0.556 and 0.66, which was similar to that of the baseline model indicating a low overlap in associated proteins of AD with PD, as well as with DLB (Fig. 5D, **Supplementary Table 22A**). However, the AUCs for predicting FTD was 0.73, which is similar to that of clinical AD (AUC = 0.796), indicating either that the current model may not be able to distinguish AD from FTD.

## Discussion

This large-scale study examined nearly 7,000 plasma proteins from 1,270 clinical AD and 2,096 cognitively normal individuals, identifying 416 proteins that were associated with clinical AD status, which were further replicated with two additional datasets. We used biomarker status based on CSF AT, amyloid imaging, and plasma ptau217 to confirm our results. We also identified the seven-protein predictive model that provided good predictive power for clinical AD and biomarker-based status, indicating that these proteins are associated with AD dementia. Our study goes beyond biomarker identification by exploring their relevance across diverse AD aspects. We compared the significant biomarkers with established AD biomarkers included CSF AT profiles, amyloid PET imaging, and plasma ptau217 levels. Additionally, we performed follow-up analyses on AD incidence, progression to symptomatic AD, and memory decline (measured by CDR-SB). We also conducted pathway and network analyses, including MEGENA, to provide deeper insights into the biological mechanisms underlying AD.

In this study, we used a three-stage approach (discovery, replication, and meta-analysis) to identity proteins associated with clinical status of AD. This approach found 456 significant aptamers (416 proteins) that demonstrated consistent directionality in discovery and replication stages and passed stringent multiple test correction in the meta-analyses. More importantly, to further validate our findings, these findings were replicated with two external datasets (ROSMAP and GNPC), supporting the validity of our findings. Of those 416 proteins (456 aptamers) identified in the study, 29% (122 proteins / 123 aptamers) have been reported to be associated nominally in previous plasma studies^15,23–27^, indicating that 71% of the findings (294 proteins / 333 aptamers) are novel. Within reported findings, 57% (70 proteins / 71 aptamers) showed a nominal association in previous plasma studies, but we report them as significant after multiple test correction here for the first time, illustrating statistical power of this study. Furthermore, of the novel proteins, 140 aptamers also have supporting evidence for association with AD risk in CSF.

In addition, we also found a significant correlation between the effect size for the proteins identified in this study and those from previous plasma studies, not only with those using SomaScan, but also Olink. Together, this supports and provides replication for our findings. However, as this study includes a very large number of samples and proteins, a total of 193 aptamers (168 proteins) were reported to be associated with AD in this study for the first time. They included SPC25, CLU, and PPBP, that were involved on signal transduction, and SPARC, NCAM1 and VEGFA implicated in endothelial pathways. Other known proteins identified in this study and part of these pathways included SMOC1, SMOC2, and ACHE, that have been reported to be associated with AD in CSF and brain in previous studies^16,29^. The lack of perfect overlap in proteins across plasma studies can be attributed to several factors, including differences in platforms (SomaScan, Olink, Mass Spec), sample sizes (most of the previous plasma studies for AD includes less than 1,500 samples and even lower number of AD cases (lower than 700)), or study design (some studies used clinical case-control status, other biomaker status and other perform survival cox regression analyses for incident AD). All of these differences add heterogeneity one to one comparison challenging. However, the fact that 122 proteins (36.52%) were reported as significant in previous studies, and, more importantly, that 204 proteins (61.1%) showed consistent directional effects, underscores the robustness and reliability of our findings.

In contrast to recent plasma proteomic studies, this study considered well-characterized AD patients. This study includes a very large cohort of cognitively normal individuals and those diagnosed with Alzheimer’s disease (AD) focused on identifying biomarkers associated with the clinical status of AD. Specifically, we analyzed 3,366 samples, comprising 1,270 individuals with AD and 2,096 cognitively normal controls. Other case-control studies have sample sizes ranging from 277 to 52,645. Among these, two notable large-scale studies—the Atherosclerosis Risk in Communities (ARIC) study and the UK Biobank (UKBB)—included more extensive samples (4,110 and 52,645, respectively). However, both studies focused on incident dementia or AD using a Cox proportional hazard model, with only 428 dementia and 691 AD cases included. Moreover, the definition of AD in the UKBB is not clearly outlined, which could introduce variability or uncertainty in the results derived from this cohort. The Atherosclerosis Risk in Communities (ARIC) study and the UK Biobank identified 38 and 5 proteins associated with memory decline, respectively^23,24^. Both studies analyzed dementia in general, which included not only AD but also other types of dementias, leading to heterogeneity and loss of power. Some of the blood samples in those studies were collected 15 to 20 years before the clinical diagnosis. Although it is known that some key AD biomarkers change years before clinical onset, not all proteins start changing that early during the disease progression^29^. Furthermore, some samples included as controls in those studies were from young individuals who may go on to develop dementia, which also impacts statistical power.

As several large-scale proteomic studies have been performed in CSF, it is important to compare the plasma results with those from CSF. To do this, we leveraged the largest CSF proteomic study performed to date, which included 2,286 CSF samples profiled using the same proteomic platform and cohort as in this study, facilitating direct comparison. We found that of around 45% of the plasma-associated proteins were also reported to be associated in CSF. On the other hand, only 8% of the CSF-associated proteins showed association in plasma, indicating that CSF has more power to identify proteins associated with AD, probably due to its close contact with brain. Another potential explanation is platform limit of detection, assuming brain proteins have low concentrations in plasma. However, this is unlikely, as the SomaScan assay was originally optimized for plasma, and similar number of proteins passed QC in both tissues. All these together suggest that proteomic signatures in CSF and plasma appear to be different, leading to a different set of proteins associated with AD depending on the tissue analyzed.

There is an open question in the field about how well blood/plasma proteomics capture brain-related processes (such as those involved in AD) and whether it is a valid biofluid to identify novel biomarkers and learn about processes implicated in neurodegeneration. Although at first glance the enriched pathways may seem to capture general processes (blood homeostasis, extracellular matrix pathways, signaling), a detailed analyses of the identified pathways and proteins clearly indicates an enrichment of endothelial proteins (ACHE, SMOC1, SMOC2, VEGFA, VEGFB, SPARC, NRCAM, NCAM1). In addition, several blood homeostasis pathways (including the plasma-novel proteins: ATP1B2, PF4, PPBP, SPARC) were identified, which may reflect vascular health. These results suggest that the pathways identified in these analyses are capturing dysregulation of the endothelia cells, which would lead BBB dysfunction and subsequent leakage of brain proteins in the blood.

The brain-related pathways included pathways related to synaptic transmission and upregulation of the GABA (gamma-aminobutyric acid) synthesis, release, reuptake, and degradation pathway. GABA is the primary inhibitory neurotransmitter in the brain, crucial for regulating neuronal excitability. One study found increased GABA in reactive astrocytes in the dentate gyrus of a mouse model, which led to tonic inhibition, a persistent reduction in neuronal activity, and memory deficiencies^38^. Another mouse study reported a bell-shaped relationship between GABA accumulation in hippocampal astrocytes and amyloidosis^39^. As another brain-relevant pathway, we found the myelination signaling pathway, which is vital for maintaining the integrity of nerve fibers, with neurofibrillary tangle formation, abnormal synaptic contraction, and activation of microglia and astrocyte release of inflammatory cytokines^40^. Taken together, our study highlights several important pathways related brain related processes that may be captured in blood due to dysfunction of the endothelia leading to BBB leakage. Some of these pathways are not a just mere reflection of the neurodegeneration but also part of the causal pathways. Several pathways (lipid metabolism, immune response < include proteins that are known part (APOE, CLU, BIN1) of the causal pathways and therefore could become potential intervention targets.

In order to learn more about AD biology and identify those processes that are part of the disease, we performed pathway analyses identifying five major super-pathways: 1) lipid metabolism; 2) immune and hemostasis 3) extracellular matrix; and 4) neuronal-related; 5) general metabolism pathways. These pathways included newly identified proteins, such as APOB, SCP2, and CRP, along with well-established AD-related proteins including CLU, APOE or BIN1, that have been identified in genetics studies, supporting that some of these pathways and processes are part of the causal pathway and not only secondary to the disease. Other known proteins identified in this study and part of these pathways included SMOC1, SMOC2, and ACHE, that have been reported to be associated with AD in CSF and brain in previous studies^16,29^. These findings corroborate previous research indicating that plasma proteomics is significantly related to lipid metabolism^23^ and the extracellular matrix^14^.

As collection of blood is considered to be minimally invasive, there is substantial interest in developing blood-based biomarkers. Plasma ptau217 shows very strong predictive power for amyloid positivity^6^, but this biomarker captures AD pathology and not overall dementia or disease status. In addition with new anti-Aβ therapies that may substantially remove fibrillar Aβ deposits as detected by amyloid-PET scan in the brain, some recent studies indicate that ptau217 decreases according to amyloid removal, even if neurodegeneration and disease progression may have not been stopped^41^. Therefore, additional non-tau and non-Aβ biomarkers are needed. For this reason, we sought to determine if the proteins identified in this study could be leveraged to create novel predictive models. Through machine learning we were able to identify a set of seven proteins that showed high AUC for clinical status and replicated in several studies (AUC > 0.72). Although this AUC may seem low, we hypothesized that this could be because around 30% of the clinical controls may have already developed AD pathology. When examining the predictive power of this model for biomarker status based on amyloid imaging, CSF AT status or plasma ptau217, the AUC was over 0.879. In addition, when testing if this model could identify those individuals that were cognitively normal at blood draw but later would progress to symptomatic AD, we found a strong association (hazard ratio = 2.71; p = 1.99×10^−4^), confirming that in fact this model can identify people with AD pathology and not only clinical status. Although we do not yet understand how Aβ therapies might alter this predictive power, this high correlation of our model with the rate of progression in CDR-SB suggests that the proteins in the model may reflect overall brain health in AD cases, not just Aβ pathology. However, additional studies are needed to confirm whether these seven proteins or combination of proteins highly correlated with these seven proteins can be used as biomarkers in a clinical setting.

In this study, we aimed to validate our seven-protein model using diverse platforms, including SomaScan, Alamar, and Olink, with data from the Knight ADRC, Stanford ADRC, BBRC, and FACE cohorts. Our experimental design was set up under head-to-head conditions to ensure the most rigorous comparison possible. The differences in AUC values between single-protein and multi- protein models across platforms were minimal. It is important to mention that we were not able to validate the model with all seven proteins, as only two proteins from the model were present in Alamar and four proteins in Olink. However, the predictive power (AUC) observed for the Alamar and Olink platforms differed only slightly from that of SomaScan. The differences in AUC values for both single-protein and multi-protein models were less than 0.07 across platforms (**Supplementary Fig. 9–11; Supplementary Table 24**). For the Alamar platform, a higher AUC was observed with only two proteins, indicating consistent predictive reliability. Additionally, for the Olink platform, the 95% confidence intervals for AUC overlapped when using four proteins. Despite this limitation, the subset of proteins available on these platforms demonstrated comparable predictive performance, further supporting the robustness of our selected proteins for accurate prediction across diverse platforms.

Another strength of the study is that we also examined the predictive performance of this model with other non-AD dementia, which is rarely done for biomarker discovery. We found that our model shows a very low overlap with PD (AUC = 0.546), and DLB (AUC = 0.661), but not with FTD (AUC = 0.73). Notably, the AUC for predicting FTD is similar to that for AD (AUC = 0.796), suggesting potential limitations in the model’s ability to distinguish between AD and FTD. Several factors may account for this, including the relatively small sample size of FTD cases (n = 32) to fully distinguish AD from FTD. Moreover, overlapping biological pathways and pathological features between AD and FTD, as well as the possibility of misdiagnoses among FTD cases, could have further influenced these results. Larger studies focusing on these groups are needed to develop disease-specific predictive models and better determine how specific the current model is for AD.

Despite the strengths of this study, several limitations should be noted. First, while we identified a robust set of proteins associated with clinical AD status, this study did not establish a causal relationship between these proteins and the disease. These findings are valuable for predicting disease status; however, further research is required to explore the causal links between the proteins and AD. Second, although we analyzed the progression to symptomatic AD, the number of incidents observed in controls was relatively small compared to previous studies^23^. Nonetheless, our study focused specifically on AD samples, offering novel insights into the progression to symptomatic AD in relation to clinical AD status, whereas previous studies considered general dementia. Third, we used multiple independent datasets (ROSMAP, GNPC, ALFA, and FACE) to replicate our findings, as well as additional SomaScan- and Olink-based published studies to further validate our findings. We found similar replicability of our findings across the three SomaScan and three Olink studies we used for comparison. Besides these efforts, we did not be able to find supportive evidence for every single proteins associated with AD in this study. The variation in the replication across studies is more likely due to several factors, including difference in platforms (SomaScan, Olink, Mass Spec), sample size (most of the previous plasma studies for AD includes less than 1,500 samples and even lower number of AD cases (691 AD cases)), or study design (some studies used clinical case-control status, other biomarker status and other perform survival Cox proportional hazard regression analyses based on incident AD or dementia). All of these differences add heterogeneity on the comparison and there is not a real one to one comparison. However, the fact that a significant number of proteins (122; 36.52%) have been reported as significant in previous studies, and more importantly that a total 204 (61.1%) were in the same direction with previous studies support the robustness of these findings. In addition, recent work by Eldjarn, *et al.*^42^ demonstrated that SomaScan assays exhibit higher precision than Olink assays, suggesting generalizability in our findings. Finally, although we identified seven proteins capable of predicting both clinical AD status and AD biomarker status, that was orthogonally validated using Alamar and Olink, further work is required to translate these findings into clinical practice. Specifically, an assay needs to be developed for these seven proteins to enable their use in clinical settings.

In summary, this large-scale plasma proteomic study identified proteins associated with AD, developed a robust prediction model that accurately predicted AD status, examined differences between predicted groups in progression to symptomatic AD and dementia, and revealed pathways and biological processes relevant to AD. These findings from extensive analysis provide the potential of plasma proteins as biomarkers for routine clinical uses in future for the early detection of AD and guiding AD treatment decisions.

## Methods

### Cohorts

Plasma samples used in this study were obtained from the Charles F. and Joanne Knight ADRC (Knight ADRC, n = 2,948), Stanford ADRC (n = 450), Movement Disorder Clinic at Washington University (MARS, n = 985), Religious Orders Study and Memory and Aging Project (ROSMAP, n = 472),Global Neurodegeneration Proteomics Consortium (GNPC, n = 6,566), ALFA at BarcelonaBeta Brain Research Center (BBRC, n = 392), and Alzheimer Center Barcelona (FACE, n = 795).

#### Knight ADRC

The Knight ADRC at Washington University School of Medicine in St. Louis has conducted prospective studies on memory and aging since 1979. These studies involved the recruitment and longitudinal assessment of community-dwelling adults over the age of 45. Knight ADRC research is approved by the Washington University Human Research Protection Office. All study participants provided written informed consent.

Annual assessments of the participants were performed by experienced clinicians using a semi-structured interview and detailed neurological examination. Interviews included the symptomatic individual and knowledgeable collateral sources in accordance with the Uniform Data Set protocol of the National Alzheimer’s Coordinating Center^43^. A clinical diagnosis of dementia is considered by study clinicians after each annual assessment, integrating results from the clinical assessment and bedside measures of cognitive function^44^. Dementia was diagnosed according to the National Institute of Neurological Disorders and Stroke (NINDS) criteria^45^ and National Institute on Aging-Alzheimer’s Association Work Group criteria for participants assessed after 2011^46^. Diagnosis of AD dementia was made in accordance with criteria developed by working groups from the National Institute of Aging and the Alzheimer’s Association^46^. Knight ADRC participants were included in this study if they had no cognitive impairments and a global clinical dementia rating (CDR) score of 0 at the time of enrollment.

A total of 2,948 participants were included in the present study (mean age 75, 58% female, 42% with one or more APOE ε4 risk alleles). Among these participants, 1,694 participants were classified as cognitively normal, 1,222 participants were diagnosed with AD, and 32 participants were diagnosed with FTD. Among 1,694 cognitively normal individuals, 83 individuals later progressed into symptomatic AD. Notably, individuals with a non-AD diagnosis at last visit or even autopsy were removed from the analyses, ensuring the prevalence of non-AD dementia in our AD cases is minimal. SomaScan 7K proteomics data generated from these participants’ samples were utilized for this study.

Plasma samples were collected in the morning without fasting. After collection, plasma samples were immediately centrifuged and stored at −80°C until they were sent for protein measurement. A detailed description of the Knight ADRC sample collection and multi-omics data available can be found elsewhere^47^. Plasma samples were collected from 1995 to 2021. Between 1998 and 2006 blood draw protocol was different than others, therefore year of sample collection was used to split the dataset into discovery and replication datasets for differential abundance analysis and predictive modeling.

In the discovery dataset (Knight ADRC 1995–1997 and 2007–2021) 1,381 participants were classified as cognitively normal, 750 participants were diagnosed with AD, and 10 participants were diagnosed with FTD. In the replication dataset (Knight ADRC 1998–2006), 313 participants were classified as cognitively normal, 472 participants were diagnosed with AD, and 22 participants were diagnosed with FTD.

#### Stanford ADRC

The Stanford ADRC cohort consists of individuals enrolled in a longitudinal observational study, including clinical dementia subjects and age-sex-matched nondemented subjects. The acquisition of plasma samples was conducted with the approval of the Institutional Review Board of Stanford University and written consent was obtained from all participants. Further details are described in Oh, *et al.*^48^.

A total of 450 participants were included in the replication cohort of the present study (mean age 70, 59% female, 30% with one or more APOE ε4 risk alleles). Among them, 402 participants were classified as cognitively normal and 48 participants were diagnosed with Alzheimer’s disease. SomaScan 7K proteomics data generated from these participants’ samples were utilized for this study.

#### Movement Disorder Clinic at Washington University

Movement Disorder Clinic at Washington University (MARS) cohort focused on participants with PD and other movement disorders. The primary objective of the study is to identify potential biomarkers of early disease progression and memory decline in individuals with PD^49,50^. Samples, including DNA, blood RNA, and CSF, are collected from these participants for research purposes.

A total of 985 participants were included in the present study (mean age 67, 42% female, 21% with one or more APOE ε4 risk alleles). Among them, 204 participants were classified as cognitively normal, 78 participants were diagnosed with DLB, and 703 participants were diagnosed with PD. SomaScan 7K proteomics data generated from these participants’ samples were utilized for this study.

### ROSMAP

The Religious Orders Study and Rush Memory and Aging Project (ROSMAP) are both community-based longitudinal cohort studies run by the Rush Alzheimer’s Disease Center, focusing on aging and Alzheimer’s disease (AD). ROSMAP comprises the Religious Orders Study (ROS) and the Rush Memory and Aging Project (MAP), both of which involve older individuals who agreed to brain donation after death^51^. The ROS study, initiated in 1994, enrolled participants from over 40 religious communities across the United States, focusing on those without known dementia at enrollment. The MAP study, which began in 1997, was designed to complement ROS by enrolling a broader population from continuous care retirement communities and individual home visits in northeastern Illinois. Both studies involve annual evaluations of participants’ physical and cognitive functions, with diagnostic assessments for conditions such as dementia, Alzheimer’s disease, stroke, parkinsonism, and depression. Upon participants’ deaths, comprehensive neuropathologic evaluations are conducted following standardized procedures, including assessments of AD pathology, cerebral infarcts, Lewy body disease, and other aging-related pathologies. Both studies were approved by an Institutional Review Board of Rush University Medical Center. All participants signed informed and repository consents and an Anatomic Gift Act.

A total of 472 participants were included in the present study (mean age 85, 74% female, 26% with one or more APOE ε4 risk alleles). Among them, 322 participants were classified as cognitively normal, and 150 participants were diagnosed with AD and confirmed with neuropathology. SomaScan 7K proteomics data generated from these participants’ samples were utilized for this study.

### GNPC

The GNPC is an initiative aimed at discovering biomarkers by integrating and expanding proteomic data from thousands of patient samples across global dementia cohorts, resulting in a comprehensive, harmonized proteomics dataset^52^. Cross-sectional and longitudinal SomaScan (SomaLogic, Boulder, CO) proteomics data were incorporated into this initiative. The GNPC successfully brought together over 40,000 samples from more than 23 different international cohorts, created a harmonized dataset that covers multiple neurodegenerative disorders, and includes approximately 300 million protein measurements. GNPC neurodegenerative disorders include AD, PD, FTD, amyotrophic lateral sclerosis ALS.

For this analysis, we used GNPC data version 1.1FF. A total of 6,566 participants were included in the present study (mean age 72, 41% female). Unlike other cohorts, we considered CDR and clinical diagnosis for defining the case and control group for this cohort. Individuals with CDR = 0 and CDR > 0 were defined as control and case groups, respectively. Absent CDR data, we relied on clinical AD diagnosis to assign case-control status. Among them, 4,833 participants were in the control group and 1,733 participants were in the case group. SomaScan 7K proteomics data generated from these participants’ samples were utilized for this study.

#### ALFA at BarcelonaBeta Brain Research Center (BBRC)

The ALFA cohort is established as a research platform to understand the early pathophysiological alterations in preclinical AD and is composed of cognitive normal individuals (between 45 and 75 years at baseline), enriched for family history of AD and genetic risk factors for AD^53,54^. CSF amyloid-β (Aβ) status was defined by the CSF Aβ42/40 ratio, and participants were classified as CSF Aβ-positive (A+) if CSF Aβ42/40 < 0.071^55^. A total of 392 participants of the ALFA (Alzheimer and Families) cohort were included in this analysis.

### FACE

Alzheimer Center Barcelona (ACE) was founded in 1995 and has collected and analyzed nearly 18000 genetic samples, diagnosed over 8000 patients, and participated in almost 150 clinical trials during its existence. This cohort encompasses a comprehensive collection of baseline samples, gathered on the day of the lumbar puncture, including matched CSF, plasma, serum, saliva, and peripheral blood mononuclear cell (PBMC) specimens. All biospecimens obtained were part of the ACE collection, registered in Instituto de Salud Carlos III (ISCIII, Ministry of Health of Spain) with the code C.0000299.

In the ACE cohort, the syndromic diagnosis of all subjects was established at the Memory Clinic of ACE (Barcelona, Spain) by a multidisciplinary group of neurologists, neuropsychologists, and social workers. We assigned to healthy controls including individuals with subjective cognitive decline diagnosis a CDR of 0, and mild cognitive impairment (MCI) individuals a CDR of 0.5. For the establishing the diagnosis, we also considered the classification of Lopez, *et al.*^56^, Petersen’s criteria, and the neuropsychological battery of ACE (NBACE)^56–64^. We used the 2011 National Institute on Aging and Alzheimer’s Association (NIA-AA) guidelines for AD diagnosis^9^. All Ace clinical protocols have been previously published^63–65^. For more details, visit www.fundacioace.com/en.

All the study’s sample collection protocols have been approved by the Clinical Research Ethics Commission of the Hospital Clinic (Barcelona, Spain) in accordance with the Declaration of Helsinki and the current Spanish regulations in the field of biomedical research. Likewise, in accordance with Spain’s Data Protection Law, all participants were informed about the study’s goals and procedures by a neurologist before signing an informed consent form. Patients’ privacy and data confidentiality were protected in accordance with applicable laws.

### Proteomics data and quality control

#### SomaScan proteomics QC

To measure the protein expression levels, a multiplexed, single-stranded DNA aptamer assay (SomaScan) developed by SomaLogic (Boulder, CO) was employed, which allowed the measurement of approximately 7,000 proteins. The SomaLogic SomaScan assay utilizes slow off-rate modified DNA aptamers (SOMAmers) to bind specific proteoforms of proteins with high specificity. In some instances, distinct aptamers are designed to target different proteoforms of the same protein. It is important to note that this technology provides an indirect measurement of proteins. The expression values reflect the binding interaction between aptamers and proteins, rather than direct protein quantification as achieved through mass spectrometry. Protein levels were reported as relative units of intensity (RFU). SomaLogic initially performed the data normalization, and subsequent, centralized quality control (QC) steps were applied to the normalized proteomics data.

We performed additional QC. We removed analytes for which 85% of the total samples failed the limit of detection (LOD) filter. We removed analytes if their scale factor deviated by more than 0.5 points from the median scale factor in any of the plates or if their coefficient of variation (CV) exceeded 0.15. At the level of individual observations, we identified outliers by expression levels (log_10_ transformed) were greater than the lower limit of the third quartile plus 1.5 times the interquartile range (IQR) or less than the upper limit of the first quartile minus 1.5 times the IQR. Outliers were assigned missing values. Finally, we measured the call-rate (the proportion of successful measurements) for each analyte and subject. During a first pass, we removed any analyte or subject with a call-rate less than 65%. After recalculating call-rates, we performed a second pass with a more stringent criterion that removed analytes or subjects with less than an 85% call-rate.

For Knight ADRC proteomic samples, 6,905 analytes and 2,948 samples remained for further analysis after following the QC process. We used principal components analyses to identify potential batch effects in the total Knight ADRC sample. When plotting the first two principal components, we observed two clusters. After further analysis, we recognized that samples with blood drawn from 1998–2006 clustered separately from other samples. To mitigate systematic bias caused by this batch effect, we divided the Knight ADRC sample by blood draw year so that one population could serve as the discovery cohort and the other as part of the replication cohort. Z-score normalization was conducted on each cluster; these values were used in subsequent analysis.

#### Technical and Biological Variance Validation

To assess and confirm the robustness of the SomaScan measurements, we examined repeated measurements of the same blood samples processed on different days (i.e., in different batches) using Knight ADRC cohort samples. The proteomic data at the Knight ADRC was generated in three proteomic datasets: Batch_1 (03/2022), Batch_2 (04/2023) and Batch_3 (05/2021). We identified 118 overlapping samples between Batch_1 and Batch_2, and 102 overlapping samples between Batch_1 and Batch_3. Sample-Level Correlation: across repeated measurements of the *same samples* in different batches, the mean Pearson correlation was 0.977 (median 0.987; **Supplementary Fig. 14**). This high correlation demonstrates excellent reproducibility and confirms that our proteomic pipeline performs consistently at the overall sample level. Protein-Level Correlation: the median Pearson correlation at the protein level was 0.702; **Supplementary Fig. 14**). Although lower than the sample-wise correlation, such results are expected in proteomic datasets where a small subset of proteins may act as outliers due to biological or analytical variations. Overall, the strong sample-level reproducibility underscores that the observed signals are robust and not predominantly driven by technical noise.

#### Alamar/NULISA proteomics QC

Protein expression levels were measured using the Nucleic acid Linked Immuno-Sandwich Assay (NULISA^™^), and protein concentrations were reported in NULISA Protein Quantification (NPQ) units. These NPQ values were normalized to account for intraplate and intensity variability, then transformed to a normal distribution using a log2-based transformation. Analytes were removed if more than 15% of the samples failed the limit of detection (LOD) filter or analytes with a coefficient of variation (CV) exceeding 15% were excluded. At the level of individual observations, we identified outliers by expression levels were greater than the lower limit of the third quartile plus 1.5 times the interquartile range (IQR) or less than the upper limit of the first quartile minus 1.5 times the IQR. Outliers were assigned missing values. Finally, we measured the call-rate (the proportion of successful measurements) for each analyte and subject. During a first pass, we removed any analyte or subject with a call-rate less than 65%. After recalculating call-rates, we performed a second pass with a more stringent criterion that removed analytes or subjects with less than an 85% call-rate.

#### Stanford Olink proteomics QC

The data we used were from Stanford ADRC cohort and QC process was performed as before^66^. Briefly, data normalization and QC can be found at https://olink.com/knowledge/documents. Data normalization and Standardization white paper, including Olink^®^’s built-in QC system, NPX calculation and definition, and the different normalization methods they used.

#### ALFA proteomic QC

Non-fasted EDTA plasma samples from the ALFA cohort were obtained following standard procedures^67^. Proteomic profiling of the 392 plasma samples was performed by SomaLogic Inc. (Boulder, CO, USA) and Olink Proteomics (Uppsala, Sweden). SomaLogic employed an aptamer-based technology (SomaScan v4.1 7K), evaluating the relative abundances of 7,289 human protein targets, quantified as relative fluorescent units (RFU). Quality control (QC) was performed by SomaLogic at both the sample and aptamer levels included corrections for hybridization variability and assay variance using hybridization controls and calibrators. Normalization across samples within a run was achieved using hybridization scale factors and median scale factors. Protein mapping to UniProt IDs and gene names was provided by SomaLogic. RFU values were log2-transformed, z-scored, and outliers were removed using interquartile range (± 1.5) thresholds.

For Olink, the Explore 3K panel (~ 3000 proteins) was analyzed using proximity extension assay (PEA) technology. Samples were randomized across plates containing manufacturer-provided intra- and inter-plate QC controls and analyzed in two rounds. Each round included 23 bridging samples representing diverse demographics and clinical groups for reference sample normalization to account for potential batch effects. Proteins were retained if values exceeded the lower limit of detection (LOD) in ≥ 85% of samples and passed QC. Raw values below the LOD were kept as provided. Proteins with > 40% missing values were excluded, while no samples were removed, as all had < 20% missing data. Duplicated assays were retained with unique names. As Normalized Protein Expression (NPX) values provided by Olink have has already been Log2, following z-score, outliers were removed using interquartile range (± 1.5) thresholds.

#### FACE proteomic QC

Plasma samples were obtained following consensus recommendations^68^, and biomaterials were stored at −80°C. The CSF Aβ40, Aβ42, total-tau, and ptau181 biomarkers were quantified by using the Lumipulse G600II automated platform (Fujirebio Europe, Göteborg, Sweden) or Innotest^®^ ELISA immunoassays (Fujirebio Europe, Göteborg, Sweden), with quality control measures in place. Cut-offs for ATN classification and the standardization of Aβ42 results for comparability across different platforms have also been established^65^. Additionally, a subset of these individuals was analysed in the SOMAscan 7k v4.1 proteomic platform (n = 1370) (SomaLogic Inc., Boulder, CO, US) as well as the Olink Explore platform analyzing over 2900 proteins (n = 508) (Uppsala, Sweden).

### Biomarker-based classification

Some level of misdiagnosis of AD can be expected by dementia clinical diagnosis, especially in early stages of disease, with some estimates as high as 30%^69^. Furthermore, the biological severity of AD does not always correspond directly to the clinical severity of the disease^69,70^. However, biomarkers offer a reliable means to identify AD pathology in individuals exhibiting cognitively impairment. Thus, we performed sensitivity analyses of biomarker-based diagnoses to further understand the pathology of AD.

The biomarker-based classification of AD in this research study was based on 1) CSF biomarkers, 2) amyloid PET, and 3) plasma ptau217. For CSF biomarkers, Aβ−42 and ptau181 were measured using the LumiPulse G platform by Fujirebio. Quantitative amyloid-PET data were acquired using AV45 and Pittsburgh compound B (PIB), and the data was normalized to reference cerebellar regions to obtain standardized uptake value ratios (SUVR) in a composite of cortical brain areas. Finally, ptau217 levels in plasma were measured using the NULISAseq platform from Alamar Bioscience (Fremont, CA). Further details are available in Ibanez, *et al.*^71^.

To identify biomarker cutoffs for CSF classification, amyloid PET imaging, and ptau217, we employed a Gaussian mixture model (GMM) approach, as previously reported^28^, using the “mclust” package (Version 6.0.0) in the R. Z-score values were calculated for each measurement to determine the cutoffs for dichotomization. These cutoffs were then used to infer the corresponding raw value cutoffs, providing biologically meaningful biomarker levels. The same approach was applied to dichotomize all four measurements.

For Knight ADRC cohort, samples with CSF-based AT status (256 AT− vs. 105 AT+), amyloid PET imaging (265 A− vs. 122 A+), and plasma ptau217 (942 T− vs. 1,217 T+) were used. For the Stanford ADRC cohort, we utilized the CSF-based AT status provided by the study, which was measured using CSF (59 AT− vs. 22 AT+).

### Differential abundance analysis

A total of 6,905 analytes were retained for the differential abundance analysis of protein levels between the AD and control (CO) groups. First, a linear regression analysis was performed with the Discovery study population with protein level as the outcome, clinical diagnosis (AD vs. CO) as the primary exposure, and age at blood draw and sex as covariates. Second, proteins with a nominal association to AD (p < 0.05) were tested in the replication study population. We used linear regression as above but added cohort as a covariate. Lastly, a meta-analysis was conducted for those proteins that were nominally significant in both discovery and replication and demonstrated consistent directionality. Effect sizes and p-values obtained from the two datasets were used for the meta-analysis using the metapro R package^72^. To address multiple testing comparisons and determine statistical significance, a false discovery rate (FDR) p-value threshold of less than 0.05 was employed using the Benjamini-Hochberg (BH) procedure.

Additionally, we leveraged other AD-related phenotypes available in the Knight ADRC to investigate their associations with protein levels. These phenotypes included amyloid PET imaging, CDR, Mini-Mental State Examination (MMSE) score, CSF biomarkers such as Aβ 42/40, total tau, and ptau181, tau PET imaging, and white matter hyperintensity volume. The number of samples used for each phenotypes varied depending on data availability. To assess the correlations between protein abundances and these AD-related phenotypes, we used the Spearman correlation coefficient. To visualize the results, we generated a heatmap plot with proteins clustered using a hierarchical clustering algorithm.

### Odds ratio (OR) analysis

Following the differential abundance analysis, we evaluated the association between protein levels and AD risk using tertile-based Odds Ratio (OR) analysis. For each of the 456 associate proteins, the highest (3rd) and lowest (1st) tertiles of protein abundance were compared using logistic regression models implemented in R with the stats package (glm function). AD status was modeled as the outcome, and protein tertile (1st vs. 3rd), age, sex, and cohort were included as covariates. The results are summarized as ORs with 95% confidence intervals.

### Sensitivity analysis

A sensitivity analysis was also performed for AD biomarker status using CSF AT status, Amyloid PET, and ptau217 status. As above, we used linear regression with protein level the outcome, biomarker group as the exposure, and age at blood draw and sex as covariates. We used effect sizes from sensitivity analysis with AD biomarkers and summary statistics from these external studies to assess the correlation with meta-analysis effect sizes from clinical diagnosis of AD. We utilized the “cor.test” function in the R.

To contextualize our findings, we conducted a comprehensive comparison between our results obtained from the clinical status-based meta-analysis and other relevant external proteomics studies. Specifically, we considered six plasma studies (Walker, *et al.*^23^, Sung, *et al.*^15^, Sattlecker, *et al.*^27^, Jiang, *et al.*^25^, Whelan, *et al.*^26^, and Guo, *et al.*^24^) and one CSF study (Ali, *et al.*^16^) in our analysis. Notably, for Walker, *et al.* and Guo, *et al.*, they employed a Cox proportional hazard regression model to identify significant proteins, we used the nature log of the hazard ratio (ln[HR]) for the correlation calculation. From these studies, we gathered summary statistics to compare with the results obtained from our research.

To compute fold-enrichment of proteins with consistent direction of effect sizes, we used the binomial distribution. This enabled us to calculate the fold-enrichment by comparing the observed number of proteins with a consistent direction of association to what would be expected by chance. Assuming the null hypothesis of no enrichment, we considered the number of available proteins for comparison and set the probability of a protein being in a consistent direction as 0.5 assuming that there is an equal chance for proteins to show either a consistent or inconsistent direction of association. The expected number of proteins in a consistent direction is then obtained by multiplying the number of available proteins by 0.5. By calculating the ratio of observed consistency to expected consistency, we determined the fold-enrichment. To assess the statistical significance of the observed enrichment, we computed a p-value using the binomial distribution. This p-value represents the probability of observing the given number of proteins (or a larger number) with a consistent direction of association, assuming the null hypothesis of no enrichment.

### Progression to Symptomatic AD

To investigate the potential association between each protein and progression to symptomatic AD, we conducted a survival analysis using the Cox proportional hazard regression model. This analysis utilized the survival R package (version 3.5–5). The Cox proportional hazard model incorporated age at plasma draw and sex as covariates along with each analyte’s expression level. For the analysis, only cognitively normal individuals (CDR = 0) at blood draw from Knight ADRC cohort were included. Subsequently, we leveraged clinical longitudinal data to identify participants that converted to AD during the follow-up period (n = 83). More detailed demographics are summarized in **Supplementary Table 13**. The time-to-event variable was calculated by subtracting the age at the blood draw date from the age at the last CDR test or the age at the onset of AD for CO and AD groups, respectively. To visualize the results in Kaplan-Meier plots, we utilized the “ggadjustedcurves” function from the survminer R package (version 0.4.9).

### Pathway Analyses

To examine the potential biological pathways associated with identified biomarkers from the differential abundant analysis, we conducted a pathway enrichment analysis using Gene Ontology (GO) (using three ontologies: Biological Process, Molecular Function, and Cellular Component) and Reactome database. We also used Qiagen Ingenuity Pathway Analysis (IPA) to identify canonical pathways, molecular networks, and biologic functions prioritized by proteins associated with AD in our study.

We used the annotation file provided by SomaLogic to map the aptamers to Entrez Gene ID. The background genes for the pathway analysis were set to include all unique genes covered by the SomaScan 7k assay, which comprised 6,101 genes. To perform the pathway enrichment analysis, we utilized the “enrichGO” and “enrichPathway“ function from the R clusterProfiler^73^ package version 4.12.0, which is available in Bioconductor.

Qiagen IPA is a bioinformatics tool that leverages the human-curated Ingenuity pathway knowledge base to link study findings to pathways, diseases, functions, and networks. IPA analysis considers effect size, direction, and p-values to implicate biologic action. Canonical pathways are well-characterized, interactive metabolic and cell-signaling pathways, and applied an FDR threshold for statistical significance. Gene and molecular interaction networks feature edges (relationships) from curate findings in the biomedical literature. These findings are also used to relate genes and proteins to biologic functions and diseases.

### Cell-type specific analysis

In this study, we performed cell-type enrichment analysis using a reference database from a study conducted by Zhang, *et al.*^74^. This reference database comprised various cell types in different human tissues derived from brain RNA sequencing data. The analysis focused on examining potential associations between identified biomarkers from the differential abundance analysis and specific cell types, including oligodendrocytes, astrocytes, endothelial, microglia/macrophage, neurons. To achieve this, hypergeometric test was employed to assess enrichment.

### MEGENA

Protein co-expression networks were constructed, and modules were identified by using the Multiscale Embedded Gene co-Expression Network Analysis (MEGENA) approach, as previously described^36,75^. Significant pathways associated with each module were identified using the same methods as described in the previous sections. The specificity of cell types in the modules were evaluated by the hypergeometric test using the human cell type gene signatures^76^ as reference following the method described in ^77,78^. Additionally, hub proteins of modules were detected using internal function in the MEGENA package^36^.

To mitigate potential impacts from covariates, protein expression data were adjusted for sex and age using a linear model, and the residuals from the regression were used to construct the co-expression network. To identify and prioritize co-expression modules that were significant in the analysis, we assessed the enrichment of our protein signature over the MEGENA modules. Proteins were divided into subsets based on positive or negative effect sizes to assess their enrichment in the MEGENA modules. The enrichment results were summarized into a ranking metric using an ensemble ranking approach^37,77^.

### Predictive model

With identified proteins from differential abundant analysis, we trained a prediction model for the clinical status of AD and CO groups. To train the prediction model, we divided the Discovery dataset into a training set and a testing set. The training set consisted of 70% of the Discovery dataset (967 CO and 525 AD). The remaining 30% of the Discovery dataset (414 CO, 225 AD) and the Replication dataset (715 CO, 520 AD) were used for testing the model.

A Lasso regression model was employed, utilizing normalized protein expression values as input with age at blood draw and sex included as covariates. The L1 regularization parameter, λ, was tuned through hyperparameter optimization using five-fold cross-validation with the “cv.glmnet” function from the glmnet R package. To reduce model complexity and prevent overfitting, we selected the highest λ value that retained 90% performance relative to the best model. The variables selected from the Lasso model were then used to retrain a logistic regression model with age and sex as covariates.

The prediction model was tested using the same cutoff for prediction and weights on biomarkers statuses, such as CSF AT status, amyloid imaging, and plasma ptau217, and other neurodegenerative diseases, including DLB, FTD, and PD. For other diseases, 100 iterations were performed to obtain robust results. During these iterations, the dataset was undersampled while maintaining a matched distribution of sex across sample groups. By undersampling, the dataset is balanced in terms of the number of cases and controls for each group to assess the model’s performance in an unbiased manner. In the case of the Knight ADRC dataset, CO samples used for training the model were excluded and undersampled for 100 iterations to match sex and sample collection year cluster distribution across sample groups.

We also compared the performance of the predictive model with known plasma AD biomarkers, specifically ptau217, ptau181, Aβ40, Aβ42, NEFL, and GFAP. The logistic regression analysis incorporated these biomarkers as independent variable, along with age and sex, to assess their predictive value for clinical status of AD. All these datasets comprised approximately 40% AD cases and 60% controls.

To evaluate the performance of the model, several metrics were calculated, including the area under the curve (AUC), accuracy, sensitivity, specificity, negative predictive value (NPV), and positive predictive value (PPV). Mean, minimum, and maximum values of these metrics were reported for other neurodegenerative diseases, providing a comprehensive assessment of the model’s performance.

### Predictive modeling for progression to symptomatic AD

To investigate the impact of predictor proteins in the trained prediction model on the progression to symptomatic AD, we conducted survival analysis using the Cox proportional hazard regression model similar to the previous analysis. However, in this analysis, samples were categorized into two groups: predicted-positive (predicted as AD) and predicted-negative (predicted as CO), based on the prediction cutoff defined during the model training phase. The predicted group was used as the independent variable of the model with other covariates. The same samples and calculation of time-to-event, as described earlier, were utilized. More detailed demographic information can be found in **Supplementary Table 13**.

This analysis was performed using the same functions as described earlier. The Cox proportional hazard model incorporated age at plasma draw and sex as covariates, along with the binarized variable representing the predicted status. The aim was to determine whether there was a difference in the rate of progression to symptomatic AD between the two predicted groups.

### Predictive modeling for memory decline (change of CDR-SB/year)

To examine the relationship between the predictive model and the progression of dementia severity, we analyzed the CDR Sum of Boxes (CDR-SB). Using the trained prediction model, we categorized the samples into two groups: predicted-positive (predicted as AD) and predicted-negative (predicted as CO). This analysis aimed to compare the rate of dementia progression in individuals and determine if there were any differences in the rate of changes in CDR-SB between the clinical statuses (AD and CO). The study utilized a total of 1,672 samples (1,002 CO and 670 AD) from the Knight ADRC, where longitudinal CDR-SB data were available.

For each sample, we selected the CDR record closest to the date of the blood draw within a 180-day window as the initial record, while the latest available CDR record was chosen as the last record. To calculate the change in CDR-SB per year for each sample, we compared the initial and last CDR-SB records. To see whether there is a significant difference in terms of the rate of progression between the two groups, we performed a Wilcoxon rank-sum test with a significance threshold of p < 0.05. Additionally, the changes in CDR-SB were visualized with respect to the years from the initial CDR test date.

## Figures and Tables

**Table 1:** Demographics information of participants for identification of biomarkers.

	Discovery		Replication	
Clinical status	CO	AD	CO	AD
**Sample size, n**	1381	750	715	520
**Age**				
mean ± SD	72.4 ± 10.5	78.1 ± 8.7	72.5 ± 9.5	76.5 ± 9.4
Range (min, max)	(27, 101)	(33, 104)	(33, 101)	(42, 98)
**Sex**				
Female	54.31%	59.22%	42.86%	54.55%
Male	45.69%	40.78%	57.14%	45.45%
**APOE genotype**				
APOE ε4+	33.1%	57.9%	25.5%	56.5%
NA	6	5	54	14
**Amyloid positivity**				
A+	632	110	143	88
A−	271	264	48	93
NA	478	376	524	339

This table summarizes basic demographic information of study participants for identification of plasma proteomic biomarkers.

Abbreviations: CO: cognitively normal; AD: Alzheimer's disease; SD, standard deviation.

**Table 2:** Demographics information of participants with other neurodegenerative diseases.

Clinical status	FTD	DLB	PD
**Sample size, n**	32	78	703
**Age**			
mean ± SD	75.5 ± 12.1	72.3 ± 9	67.1 ±9.9
Range (min, max)	(54,96)	(55,95)	(31,92)
**Sex**			
Female	16	25	262
Male	16	53	441

This table summarizes basic demographic information of study participants with other neurodegenerative diseases.

Abbreviations: DLB: dementia with Lewy bodies; FTD: frontotemporal dementia; PD: Parkinson's disease; SD, standard deviation.
